# Statistical Testing of Shared Genetic Control for Potentially Related Traits

**DOI:** 10.1002/gepi.21765

**Published:** 2013-11-05

**Authors:** Chris Wallace

**Affiliations:** ^1^ JDRF/Wellcome Trust Diabetes and Inflammation LaboratoryDepartment of Medical Genetics, NIHR Cambridge Biomedical Research Centre, Cambridge Institute for Medical Research, University of CambridgeCambridgeUnited Kingdom

**Keywords:** colocalisation, GWAS, genetic association, causal variants

## Abstract

Integration of data from genome‐wide single nucleotide polymorphism (SNP) association studies of different traits should allow researchers to disentangle the genetics of potentially related traits within individually associated regions. Formal statistical colocalisation testing of individual regions requires selection of a set of SNPs summarising the association in a region. We show that the SNP selection method greatly affects type 1 error rates, with published studies having used methods expected to result in substantially inflated type 1 error rates. We show that either avoiding variable selection and instead testing the most informative principal components or integrating over variable selection using Bayesian model averaging can help control type 1 error rates. Application to data from Graves' disease and Hashimoto's thyroiditis reveals a common genetic signature across seven regions shared between the diseases, and indicates that in five of six regions associated with Graves' disease and not Hashimoto's thyroiditis, this more likely reflects genuine absence of association with the latter rather than lack of power. Our examination, by simulation, of the performance of colocalisation tests and associated software will foster more widespread adoption of formal colocalisation testing. Given the increasing availability of large expression and genetic association datasets from disease‐relevant tissue and purified cell populations, coupled with identification of regulatory sequences by projects such as ENCODE, colocalisation analysis has the potential to reveal both shared genetic signatures of related traits and causal disease genes and tissues.

## Introduction

In recent years, genome‐wide association studies (GWAS) have facilitated a dramatic increase in the number of genetic variants associated with human disease and other traits such as gene expression. Understanding the means by which these variants exert their effect will aid the design of the detailed functional followup studies already underway. Although the causal variants are not commonly known, multiple traits have been mapped to the same genetic loci, raising the possibility that the same variants affect multiple traits either directly or with one trait mediating the other. For example, genetic susceptibility to type 2 diabetes across 12 loci appears mediated by the genetic influence on body mass index [Li et al., 2011]. Within individual loci, researchers are examining the genetic association signals from pairs of traits in parallel, with similar results interpreted as evidence that the two traits may colocalise, or share a common causal variant. These traits may be eQTL signals across two or more tissues [Dimas et al., 2009; Fairfax et al., 2012], eQTL and disease signals [Nica et al., 2010; Wallace et al., 2012] or two or more diseases [Cotsapas et al., 2011]. Distinguishing cases where related diseases share a common causal variant *vs*. those where neighbouring but distinct variants appear to underlie disease risk in a region will aid identification of cross‐disease and disease‐specific mechanisms. In addition, comparison of disease and eQTL data has the potential to reveal both the likely disease causal gene in regions where a number of candidate causal genes exist, and the relevant tissue type where tissue‐specific eQTLs exist. However, dependence between genotypes at neighbouring SNPs, caused by LD, means that determination of colocalisation is not obvious, as there may exist distinct but neighbouring causal variants for each trait that are mutually associated.

When these traits are measured in the same individuals, it is possible to use conditioning to determine whether one trait mediates the other [Li et al., 2011]. For example, if both body mass index (BMI) and type 2 diabetes (T2D) have been linked to a SNP, then when including BMI and the SNP as explanatory variables for T2D, BMI but not the SNP should show association if BMI is a mediator. However, when the traits are measured in distinct samples, or when two traits may share a common causal variant without one mediating the other, most researchers have approached the task of looking for colocalisation either by examining by eye the association signals across a set of common SNPs in the two datasets [Dubois et al., 2010] or by testing for evidence of residual association in their available dataset conditional on the most associated SNP in the other [Nica et al., 2010]. When full data for both traits are available, colocalisation may be tested by examining whether coefficients from regressions of each trait against two or more SNPs are proportional, as they should be if those SNPs jointly tag a common causal variant [Plagnol et al., 2009; Wallace et al., 2012].

We show here that naïve application of both conditional and proportional colocalisation tests may result in substantially inflated type 1 errors, and explore reasons for that inflation. The inflation cannot be easily resolved for conditional tests, but we demonstrate two alternative approaches for proportional testing that result in unbiased inference. Finally, we apply these methods to colocalisation testing of 13 regions shown in Supplementary Table S1 that have been associated with one or both of the autoimmune thyroid diseases, Graves' disease (GD) and Hashimoto's thyroiditis (HT), using previously published dense genotyping data [Cooper et al., 2012].

## Methods

### Approaches to Colocalisation Testing

We begin by introducing some notation and setting out the details of the existing approaches to colocalisation testing that are explored in this paper. Assume two traits, *Y* and $Y^{\prime }$, have been measured in distinct samples and evidence exists for association of both traits to some genetic region. Let the region be covered by *p* SNPs genotyped in both samples, with the genotype matrices denoted by $X=\left(X_{1},...,X_{p}\right)$ and $X^{\prime }=\left(X_{1}^{\prime },...,X_{p}^{\prime }\right)$ , respectively. Conditional approaches begin with identifying the most strongly associated SNPs for *Y* and $Y^{\prime }$, SNPs *k* and $k^{\prime }$, say, then examine whether there is any evidence for association between *Y* and SNP *k* conditional on SNP $k^{\prime }$. The null hypothesis is therefore

(1) H0 cond :Y⊥Xk|Xk′.

Concerned that LD would make interpretation of the conditional test difficult, Nica et al. [2010] extended the conditional method as follows. For every SNP *j* generate residuals $R_{j}$ from a regression of *Y* against $X_{j}$ and test the correlation of $R_{j}$ and $X_{k}$ using Spearman's rank correlation test, generating *p* values $P_{j}$. The evidence against the null hypothesis (1) is then measured by the rank of $P_{k^{\prime }}$ in the empirical distribution, $\left[P_{j}\right]$, generated. This effectively compares the *P* value at the test SNP *k* conditional on SNP $k^{\prime }$ to that conditioning on all other SNPs in the region. However, note that because this method summarises evidence for colocalisation by a rank only, there is no statistical inference attached. Thresholds for interpreting ranks would be expected to depend on SNP density and LD patterns.

The proportional approach frames the null hypothesis differently. A set of *q* SNPs are chosen that are deemed somehow to jointly be good predictors of one or both traits. Regressing *Y* and $Y^{\prime }$ against these columns of *X* and $X^{\prime }$ respectively produces estimates, $b_{1}$ and $b_{2}$, of regression coefficients ***β***_1_ and ***β***_2_, with variance‐covariance matrices $V_{1}$ and $V_{2},$ respectively. Since sample sizes are large, the combined likelihood may be closely approximated by a Gaussian likelihood for $\left(b_{1},b_{2}\right)$, assuming $V_{1},V_{2}$ are known and that $Cov(b_{1},b_{2})=0$. Assuming equal LD in the two cohorts, i.e., that the correlation structure between the SNPs does not differ, Plagnol et al. [2009] show that the regression coefficients should be proportional and proposed testing for a shared causal variant by testing the null hypothesis

H0 prop :β1∝β2,

i.e., $\beta_{1}=\frac{1}{\eta }\beta_{2}=\beta$. The chi‐squared statistic

(2) $$T{\left(\eta \right)}^{2}=u^{T}V^{-1}u∼\chi^{2}$$

is derived from Fieller's theorem [Fieller, 1954], where $u=(b_{1}-\frac{1}{\eta }b_{2})$ and $V=V_{1}+\frac{1}{\eta^{2}}V_{2}$. If η were known, $T{\left(\eta \right)}^{2}$ would have a χ^2^ distribution on *q* degrees of freedom. Plagnol et al. take a profile likelihood approach and replace η by its maximum likelihood estimate, $\hat{\eta }$, which also minimises $T{\left(\eta \right)}^{2}$. Asymptotic likelihood theory suggests that $T{\left(\hat{\eta }\right)}^{2}$ has a χ^2^ distribution on q−1 degrees of freedom. Alternatively, Wallace et al. [2012] take a Bayesian approach. They begin by reparametrising the likelihood in terms of $\theta =\tan^{-1}\left(\eta \right)$ and rewriting the null hypothesis as

H0 prop :β1=βcos(θ);β2=βsin(θ).

This allows calculation of the posterior distribution of θ, $P(\theta |b_{1},b_{2})$, assuming uninformative priors for θ and ***β***. Inference is based on posterior predictive *P* values

(3) $$\int \;T^{*}\;\left(\theta \right)P\left(\theta |b_{1},b_{2}\right)\;d\theta$$

where $T^{*}\;\left(\theta \right)$ is the *P* value associated with T(tan(θ)). Full mathematical details are given in the supplementary material of Wallace et al. [2012], but it is worth revisiting here the justification of a flat prior for θ. If $\beta_{1},\beta_{2}$ were univariate with Gaussian priors and mean 0, then $\tan\left(\theta \right)=\frac{\beta_{1}}{\beta_{2}}$ would have a Cauchy(0, *k*) prior where *k* is the ratio of the prior variances of β_1_ and β_2_. Thus, it seems an appropriate form to consider for the prior for θ in the multivariate case. *k* is unknown, but Wallace et al. [2012] found that varying *k* had a negligible effect on the posterior predictive *P* value for the sample sizes common in GWAS and eQTL studies (100s to 1,000s of subjects) and we have set k=1, implying a uniform prior for θ, for all analyses in this paper.

Posterior predictive *P* values have a somewhat different interpretation than and appear conservative in comparison to standard *P* values [Meng, 1994; Rubin, 1984]. However, they avoid assuming the log‐likelihood for η is approximately quadratic near its maximum that is not always the case. In practice, Wallace et al. [2012] found standard and posterior predictive *P* values to be almost identical in large samples.

H0 prop  is not the same as H0 cond  as it does not explicitly condition on the most associated SNPs, but is a general property expected to be true of any pair of traits that share a common causal variant. While a shared causal variant should imply H0 prop  is true at any pair of SNPs, and that H0 cond  is true if $k=k^{\prime }$ is the causal variant, the reverse is not the case as it is possible that two traits have distinct causal variants in complete LD. Thus, failure to reject the null hypothesis indicates only that the data are consistent with a shared causal variant.

Note that colocalisation testing may be applied equally to case control data (using logistic regression), expression data (using linear regression) or to compare case control results against expression results for a specific gene. Most commonly, this last approach might be applied in turn to all genes with a known eQTL signal in the neighbourhood of the disease association signal. However, it is assumed that $cov(b_{1},b_{2})=0$, meaning that case control studies may only be compared if they do not share a common control group.

### Choice of SNPs for Proportional Colocalisation Testing

The choice of SNPs for colocalisation testing will be shown in this paper to have a considerable influence on the type 1 error rate of colocalisation tests. The aim of selecting the most informative subsets of SNPs for proportional colocalisation testing is to minimise the degrees of freedom of the test, and hence maximise power. However, unless independent data are available for variable selection, this increase in power comes at a cost to type 1 error rate control as shown above. In this paper, we propose two methods for avoiding this problem.

#### Summary of Genetic Variation by Principal Components

If the region of interest displays strong LD, a modest number of principal components (PCs) are generally required to capture most of the SNP variation (Supplementary Fig. S4) and   we can use a subset of the most informative components for colocalisation analysis. Because PCs are by definition uncorrelated, and because the selection is not based on their relationship to the traits of interest, the estimated coefficients at any such subset are unbiased. To allow PC analysis of two datasets, we first form a combined genotype matrix, center and scale each SNP, and then define the principal components. Colocalisation testing is performed using the projection of the data onto the transformed basis for the most important components. The optimal choice of threshold defining the ‘most important’ components is not obvious, and we explore that in our simulations.

#### Bayesian Model Averaging

Alternatively, we may combine the ideas of Bayesian model averaging (BMA) [Viallefont et al., 2001] and posterior predictive *P* values, to treat the model describing the joint association itself as a nuisance parameter, and average the *P* values not just over the posterior for η, but also over the posterior for all SNP selection models. Analogous to equation (3), posterior predictive *P* values are therefore defined by

(4) $$\mathrm{ppp}=\sum_{m\in M}p^{*}\;\left(m\right)P\left(m\right)$$

where M is the set of models under consideration, $P^{*}\;\left(m\right)$ is the colocalisation testing *P* or ppp value under the SNP model *m*, and P(m) is the posterior probability of model *m* given the data and under the assumption that one of M is the true model. To minimise the degrees of freedom of the test, we explore all two SNP models and, in the absence of any independent evidence to favour one SNP over another, we assume the prior is evenly spread over the set of models. Approximating the posterior probabilities by means of the Bayesian Information Criterion approximation [Hoeting et al., 1999; Schwarz, 1978] and discarding highly improbable models at the outset, this could be done without excessive computational burden (see Supplementary Material for full details).

Both the PC and BMA approaches are available in our R [R Development Core Team, 2010] package, coloc, available from the Comprehensive R Archive Network (http://cran.r‐project.org/web/packages/coloc).

### Simulation

We used simulation to demonstrate the effects of variable selection on the power and type 1 error rate for colocalisation testing. Full details are given in Supplementary Material. Briefly, we sampled, with replacement, haplotypes of SNPs with a minor allele frequency of at least 5% found in phased 1000 Genomes Project data [Consortium et al., 2012] across all 49 genomic regions outside the major histocompatibility complex (MHC) that have been identified as type 1 diabetes (T1D) susceptibility loci to date, as summarised in T1DBase [Burren et al., 2011]. These represent a range of region sizes and genomic topography typical of GWAS hits. We excluded the MHC region that is known to have high variation, strong LD and exhibits huge genetic influence on autoimmune disease risk involving multiple loci and hence requires individual treatment in any GWAS [Nejentsev et al., 2007].

Using a single ‘causal variant’ SNP chosen at random, we sampled case and control haplotypes according a multiplicative disease susceptibility model with relative risks of ranging from 1.1 to 1.3 to represent GWAS data. To simulate a quantitative trait, and to extend our exploration to two causal variants in each trait, we selected one or two ‘causal variants’ at random, and simulated a Gaussian distributed quantitative trait for which each causal variant SNP explains a specified proportion of the variance. To reflect our expectation that this test will be applied in cases in which some nominal association to a region has already been established, we discarded datasets in which all single SNP association $P>10^{-4}$. We either used all SNPs or the subset of SNPs that appear on the Illumina HumanOmniExpress genotyping array to conduct colocalisation testing to reflect the scenarios of very dense targeted genotyping *vs*. a less dense GWAS chip. All analyses were conducted in R [R Development Core Team, 2010] using the coloc package for proportional colocalisation testing.

### Colocalisation Testing for Autoimmune Thyroid Disease

An association study of the autoimmune thyroid diseases GD and HT has recently been completed using the Immunochip for genotyping, which provides dense coverage of regions of the genome previously associated with autoimmune disease [Cooper et al., 2012]. The paper presented a total of 2,285 Graves' disease cases, 462 Hashimoto's disease cases and 9,364 controls. We split the controls randomly into two groups of size 4,682, and analysed each of the 13 regions reported to be associated with one or both diseases [Cooper et al., 2012]. Missing data were rare, but regression models require complete genotyping data. We therefore imputed missing genotypes by means of multiple regression, as implemented in the R package snpStats [Clayton and Leung, 2007]. We conducted proportional colocalisation analysis using the the two alternative methods set out above. For the PCs approach, we used components that captured at least 90% of the observed genetic variation. For the BMA approach, we averaged either over the universe of all possible two SNP models or that of all three SNP models.

## Results

### Naive Application of Colocalisation Tests Leads to Biased Inference

**Figure 1:**
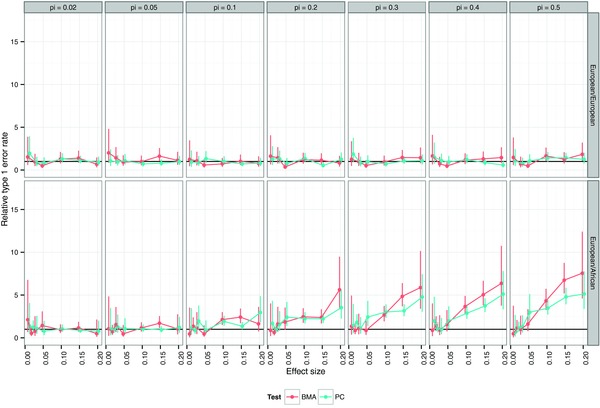
Type 1 error rates in naive colocalisation testing. A nominal type 1 error rate of 5%, shown by a dashed line, is consistently exceeded using conditional colocalisation testing conditioning on either the most associated SNP for the other trait (C1) or the common causal SNP that is only possible in simulated complete genotyping data (C2). Proportional colocalisation testing tends to exhibit lower type 1 error rates, but the excess can still be substantial when using the most strongly associated SNPs in each dataset (P1); the union of lasso variable selection in each dataset (P2) or a two stage lasso variable selection (P3) as previously described [Wallace et al., 2012]. In contrast , type 1 error rates are well controlled for proportional testing using principle components that capture 85% of the genetic variation (P4) or within a Bayesian Model Averaging approach to variable selection (P5), even appearing conservative for small effect sizes. Note that Bayesian Model Averaging was not examined in the complete genotyping scenario due to computational burden. The X axis shows the relative risk of disease (RR) with columns divided according to the number of cases and controls in a case‐control dataset. Type 1 error rates were calculated by comparing two case‐control datasets of equal sample and effect size, simulated to share a common causal variant.

The choice of SNPs to use for testing can induce bias for two reasons. First, selecting the ‘most associated’ SNP on the basis that the evidence for its association is strongest amongst all SNPs tested does not guarantee either that it is the causal SNP or even the best proxy. Random variation and LD mean that evidence for association may peak at an alternative SNP even when the causal SNP is included in the genotyping panel, a bias that is more pronounced for weaker effects and smaller sample sizes (Supplementary Fig. S1). Second, although it is well known that regression coefficients are unbiased estimates of population effects, this property does not hold after variable selection [Miller, 1984], an effect that has been referred to as ‘Winner's curse’ in genetics [Gring et al., 2001; Lohmueller et al., 2003]. Choosing SNPs on the basis of their significance or some other measure of strength of association induces a bias away from the null—i.e., coefficients of selected SNPs are expected to overestimate the true effect—and again the effect is more pronounced for smaller sample sizes and weaker effects (Supplementary Fig. S2). These two biases mean that, in conditional testing, there is likely to be some residual association between the phenotype *Y* and the remaining genetic markers after conditioning on the selected SNP $k^{\prime }$ because (1) the conditioning SNP $k^{\prime }$ may not capture all the true association and (2) the estimated effect at the tested SNP *k* tends to be an overestimate. The result is very poor control of the type 1 error rate (Fig. 1, track C1). Conditioning on the common causal variant rather than the most associated SNP (which is only possible in simulation studies) reduces the bias by removing the SNP selection problem, but does not eliminate it due to the overestimation of effect size (Fig. 1, track C2).

As seen in Supplementary Fig. S3, Nica's score tends towards to 1 for traits that share a causal variant and is uniformly distributed on [0, 1] for distinct unlinked causal variants. Its distribution is increasingly skewed towards 1 as the LD between distinct causal variants increases. This makes sense if one considers that the case of two distinct variants in some LD lies partway between the extreme cases of distinct unlinked causal variants and a single common causal variant, which is equivalent to distinct causal variants in complete LD. The effect of using the most associated SNPs for testing compared with using the true causal SNPs is to reduce the skew towards higher rank scores as the *r*^2^ between variants increases. Thus, Nica *et al*.'s extension [Nica et al., 2010] may prioritise the likely colocalisation signals within a set, but as it avoids formally testing a null hypothesis, and because the scale against which to interpret the rank score is likely varies according to effect size, it does not provide a means to assess evidence for or against colocalisation at a given locus of interest.

For the proportional approach, two strategies have been applied. Either colocalisation has been tested using the pair of SNPs *k* and $k^{\prime }$ defined above [Plagnol et al., 2009] or a lasso approach, where SNPs are first selected in a lasso for one trait, and then additional SNPs are selected in a further lasso for the other trait [Wallace et al., 2012]. However, as shown by Miller [Miller, 1984], any variable selection method must induce bias in the estimated coefficients ($b_{1}$, $b_{2}$) if the estimation occurs in the same dataset as the selection. We show here that neither method maintains control of the type 1 error rate (Fig. 1, tracks P1, P2 and P3), although the bias is less extreme than for conditional testing. The two‐step lasso selection does reduce bias compared to independent lasso selection in the two datasets, but, perhaps counter‐intuitively, leads to greater bias than simply testing the pair of most associated SNPs $\left(k,k^{\prime }\right)$ when only tagging genotypes are available and effect sizes are large (relative risk ∼ 1.3). This is because, in this situation, lasso may select SNPs that are apparently weakly associated (either truly or through random noise) at which, as demonstrated in Supplementary Fig. S2, effect estimates are more strongly biased.

### Alternative Approaches to Proportional Testing

We propose two alternative approaches to proportional testing, either of which can help control type 1 error rates.

#### Principal Components

The first is to use a set of principal components that summarise the genetic variation in the region, instead of individual SNPs. However, it is not obvious how many components are required. As more components are selected, more information about the genetic variation in a region is captured, and hence we are more likely to accurately capture the signal of any causal variants. However, successive components add decreasing amounts of information whilst still adding another degree of freedom. At some point the negative effect of increasing degrees of freedom will outweigh the positive effect of increasing information, and we were concerned that the optimal test may depend heavily on the threshold used to determine the number of components selected. Instead, power seemed broadly acceptable once components capturing 70–90% of variation were selected (Supplementary Fig. S5). In our 49 test regions, 70% of the variance could be captured by selecting an average of 7 (range 2–18) or 9 (range 3–44) components under a tagging or complete genotyping approach.

**Figure 2:**
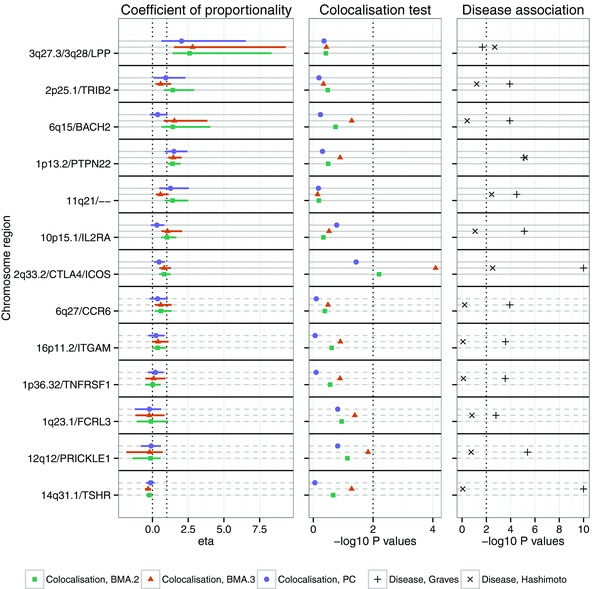
Power for proportional colocalisation analysis using PC or BMA approaches. The theoretical maximum power (Max) is calculated using the two causal variants (which are known in simulated data) and show that the predominant determinant of power is the *r*^2^ between the variants, with power decreasing as LD increases. When the causal variants are not known, power decreases under either a PC or BMA approach. The X axis shows the maximum *r*^2^ between the causal variants, i.e., *r*^2^ has been categorised into five groups: [0, 0.2], [0.2, 0.4], [0.4, 0.6], [0.6, 0.8], [0.8, 1.0]. *N* is the number of cases and controls in a case‐control dataset with relative risk of disease RR. Power is shown for comparing two case‐control studies with equal sample numbers and effect sizes (solid lines) or for comparing a case‐control study to an eQTL study of 1,000 samples where the causal variant explains 30% of the variance of the expression. The PC approach was implemented by selecting the smallest subset of components that captured 85% of the genetic variance. We considered only tagging genotype scenarios to reduce computation time.

We found type 1 error rates were controlled across the range of thresholds explored, but show for illustrative purposes the results when we fixed the threshold at ⩾90% of genetic variation (Fig. 1). We examined power to detect departure from colocalisation using simulations in which the causal variants are distinct for two traits but placed no restrictions on the LD between these variants. We first examine the theoretical maximum power of the test by testing the two causal variants themselves, which are known in a simulation study but not in real data (Fig. 2). As might be expected, power increases with sample size and effect size, but is negatively correlated with the *r*^2^ between the causal variants, and is maximum when the two are completely unlinked ($r^{2}=0$). When using PCs, the power is reduced reflecting the loss of information in not knowing these causal variants, but the loss is greatest for complete genotyping scenarios, suggesting that we may be selecting too many components in the case of complete genotyping and emphasising the difficulty in choosing one optimal threshold for all studies and regions.

#### Bayesian Model Averaging

Our second proposed approach is to test the proportional null hypothesis using all possible two SNP models. The resulting *P* values may then be averaged, weighting by each model's posterior probability, in a Bayesian model averaging framework and generating a posterior predictive *P* value. Because of its computational burden for simulations, we only consider the BMA approach under a tagging genotyping scenario. This demonstrates good control of the type 1 error, even tending to be mildly conservative, as has previously been reported when posterior predictive *P* values are interpreted similarly to standard *P* values [Meng, 1994]. Despite the slightly more conservative type 1 error rates, the BMA approach appears more powerful than the PCs approach (Fig. 2), which presumably reflects the greater degrees of freedom required for the PCs approach.

### Sensitivity to the Assumption of Equal Linkage Disequilibrium

**Figure 3:**
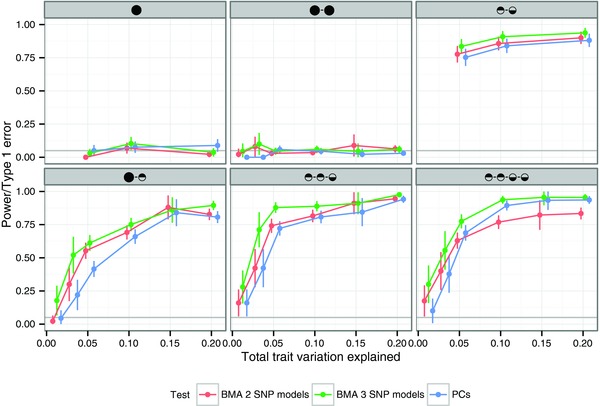
Departure from assumption of equal LD structure. Each plot reflects simulations in which a single, common causal variant explains a fixed proportion of variance of two quantitative traits, shown on the x axis, each available in a sample of 1,000 individuals. In the top row, all haplotypes in the first dataset and (1−π) of the haplotypes in the second dataset were sampled from the European CEU, GBR and FIN populations, with the remaining π in the second dataset from the alternate European TSI and IBS populations. In the bottom row, we used the same strategy but sampling either from all European populations (CEU, GBR, FIN, TSI and IBS) or from a mixture of these European populations and the African ASW, LWK and YRI populations. The y axis shows the relative type 1 error rate—the ratio of the estimated rate for the given scenario and the estimated rate for the equivalent scenario with no mixing. Because these are ratios, there is rather less certainty than for other plots and 95% confidence intervals calculated by means of the delta method are shown for each point. Analysis was conducted by proportional testing using either a PC approach with number of principal components selected to capture 90% of genetic variation or a BMA approach, averaging over the space of either all possible two SNP models. We considered only tagging genotype scenarios. CEU=Utah Residents (CEPH) with Northern and Western European ancestry; GBR = British in England and Scotland; FIN = Finnish in Finland; TSI = Toscani in Italia; IBS = Iberian population in Spain; ASW = Americans of African Ancestry in SW USA; LWK = Luhya in Webuye, Kenya; YRI = Yoruba in Ibadan, Nigeria.

The proportional colocalisation test assumes identical patterns of LD in the two datasets so that the effect of a shared causal variant is proportional across any set of SNPs. To explore its sensitivity to this assumption, we considered sampling haplotypes for one dataset from a subset of European populations, and for the other dataset from a either a mixture of European populations or a mixture of European and African populations. As might be expected, for strongly admixed datasets, the control of type 1 error rate is lost, with type 1 error rates up to eightfold that seen under the case of no mixing (Fig. 3). However, it is perhaps surprising that the effect of mixing between two European populations, or mixing very small proportion of African haplotypes (∼5%) into a mainly European population, is barely detectable at the sample sizes of 1,000 used, and indicates that the method is robust to small departures from the assumption of equal linkage disequilibrium. Of course, as with any genetic analysis, it remains sensible not to rely on this property, but to formally examine the evidence for population structure and exclude obviously outlying samples.

### The Case of Multiple Causal Variants

**Figure 4:**
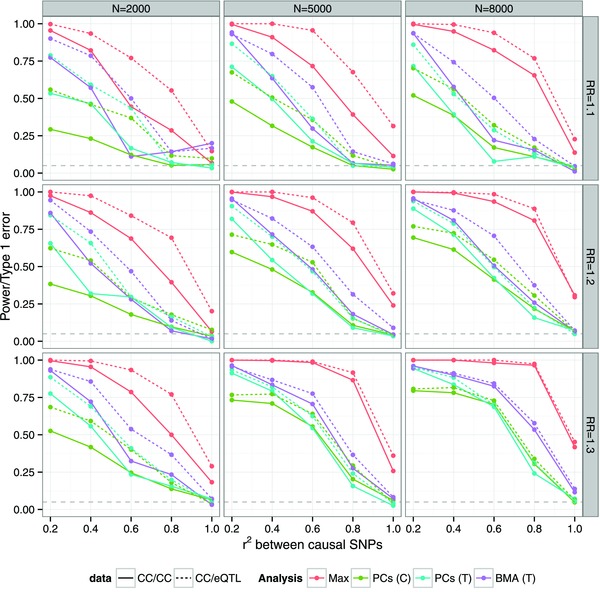
Effect of more than two causal variants. Each plot reflects simulations in which causal variants in total explain a fixed proportion of variance of two quantitative traits, shown on the x axis, each available in a sample of 1,000 individuals. The total number of causal variants is shown by the number of circles above each plot, with full circles indicating a causal variant shared by both traits and half shaded a causal variant associated with one trait or the other. Analysis was conducted by proportional testing using either a PC approach with number of principal components selected to capture 90% of genetic variation or a BMA approach, averaging over the space of either all possible two SNP or three SNP models. We considered only tagging genotype scenarios.

So far, we have only considered the case of a single causal variant for each trait. But the proportional test makes no assumption about the number of causal variants, only that their effects are proportional. Figure 4 shows that in the case of eQTL data with two shared causal variants, having equal effects on each trait, type 1 error rates are still controlled. It has been reported that genes may exhibit a common cross‐tissue eQTL, located proximal to the gene, as well as distinct tissue‐specific eQTLs in more distal locations [Brown et al., 2012]. We were therefore interested to explore the case where our two traits share one causal variant, but one or both are also under the influence of additional, distinct variants. Testing a single hypothesis of colocalising *vs*. not colocalising variants cannot capture the complexity of this situation, but it is instructive to explore the test's expected behaviour in order to allow proper interpretation with real data where the number, and sharing configuration of causal variants is unknown. Figure 4 shows that, under a tagging genotyping scenario, the proportional test tends to reject the null of colocalisation in the case of any distinct causal variants, even in the presence of an additional shared variant, although with slightly less power than when there is no shared variant.

### Varying the Number of SNPs in Bayesian Model Averaging Models

So far, also, we have assumed that it was enough to consider only the universe of two SNP models when applying our BMA approach. The motivation for this was that a two SNP model leads to a one degree of freedom test, and might therefore be expected to maximise power. We examined the effect of averaging over either all two SNP or all three SNP models in the context of the above multiple causal variant simulations. This shows that type one error rates are similar, that power is similar for two SNP models, but that power can be increased by averaging over three SNP models compared to two SNP models, particularly when there are really three or more distinct causal variants (Fig. 4).

### Application to Colocalisation Testing of Autoimmune Thyroid Diseases

Existing evidence suggests that a single locus may contain variants that predispose to any one of multiple diseases, e.g., the nonsynonymous C1858T SNP in *PTPN22* is associated with rheumatoid arthritis and T1D [Barrett et al., 2009; Stahl et al., 2010], or distinct variants that predispose to different diseases, e.g., distinct variants in *IL2RA* are associated with T1D and multiple sclerosis [Maier et al., 2009; Martin et al., 2012]. We used the proportional colocalisation approach outlined above to examine the disease signals for the autoimmune thyroid diseases HT and GD from a recent dense genotyping study [Cooper et al., 2012].

We first examined the seven regions where a significant single SNP effect has been identified in both diseases, i.e., at study‐wide significant levels for GD ($P<1.1\times 10^{-6}$, a permutation derived threshold specific to this study) and at a nominal significance threshold of P<0.05 for HT. Six of these display no evidence against colocalisation (all posterior predictive P>0.01), the exception being 2q33.2/*CTLA4*/*ICOS* in which the ppp value for the BMA approach is $6\times 10^{-3}$ (two SNP models) or $8\times 10^{-5}$ (three SNP models). In this region, the profile of the single SNP *P* values do differ (Supplementary Fig. S6), but it would require larger sample sizes to confidently conclude that the two diseases have different causal variants in this region given the number of tests completed.

**Figure 5:**
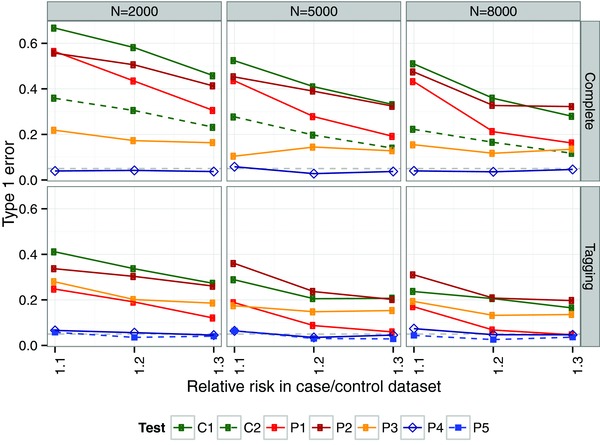
Colocalisation analysis of Graves' and Hashimoto's diseases. Regions are labelled by chromosome and likely candidate gene(s) and arranged so that the top seven regions showed marginally significant association with both GD and HT (P<0.05) and the bottom six with just GD in the published single SNP analysis [Cooper et al., 2012]. The left panel shows the estimate of the coefficient of proportionality, η, for the estimate of the ratio of effect sizes in HT compared to GD, and its 95% credible interval, calculated using either the PCs approach (PC), or BMA averaging over two SNP models (BMA.2) or three SNP models (BMA.3). The middle panel shows the the evidence against colocalisation using either the $-\log_{10}\left(P\right)$ value (PCs) or the posterior predictive $-\log_{10}\left(P\right)$ (BMA). The right panel summarises the evidence for association of the region with each disease using $-\log_{10}\left(P\right)$ values for the association analysis of Graves' and Hashimoto's using the selected principal components for the PCs approach. The $-\log_{10}$ scale has been truncated at 10 so that more extreme *P* values are displayed at $-\log_{10}\left(p\right)=10$. For the PC approach, testing was based on the smallest subset of components that captured 90% of the genetic variance.

The coefficient of proportionality, η, can be usefully interpreted when analysing two diseases. Two values of particular interest are η=0 that would indicate no effect in HT given an effect in GD and η=1 that indicates equal effects in each disease. In most of the seven regions, the credible interval for η includes 1 (Fig. 5), the exceptions being 2q33.2 and 10p15.1, where it ends just below 1 using a PCs approach, and 3q27.3/3q28/*LPP* where it starts just above 1 on either BMA approach.

Turning to the the six regions where there is evidence of association in only GD, we do not expect to see any departure from the null of colocalisation, without evidence of association to both traits, and indeed all posterior predictive P>0.01. However, our estimate of η helps infer whether this reflects a lack of power or genuine absence of association for HT. We evaluated the credible intervals for η in each region and across four of the six regions, the credible interval for η includes 0, whilst in only one does the credible interval include 1 for all approaches. In 14q31.1/*TSHR*, the credible interval ends just *below* 0 for the BMA approaches, whilst for 6q27/*CCR6*, it starts just above 0 and includes 1 for all methods. *TSHR* represents the primary autoantigen in hyperthyroidism of GD [Brand and Gough, 2010], so is unlikely to be involved in HT.

## Discussion

There are two sources to the bias in colocalisation testing presented above. The problem of variable selection is well studied in statistics generally [Miller, 1984] but has perhaps been neglected in statistical genetics, where the aim has been to detect convincing association to a region, rather than pinpoint the causal variant, particularly as most datasets to date have included an incomplete selection of variants in any region. Selecting SNPs that do not fully capture the trait association will affect conditional colocalisation testing because some residual association must remain after conditioning. On the other hand, it should not bias proportional testing as the aim there is to test for proportionality of effect size rather than evidence of residual effect. This may explain the substantially higher error rates for naive conditional testing *vs*. naive proportional testing seen in Figure 1.

The bias in effect size estimates affects both methods, however. In genetics, we are familiar with ‘Winner's curse’, which causes effect estimates examined conditional on the value of the associated *P* value to be biased away from the null. Some attempts to correct this effect size bias have been made, either by modelling a selection procedure defined as a single SNP exceeding a predetermined level of significance [Zollner and Pritchard, 2007], or by bootstrapping that can in theory account for the full selection strategy [Sun et al., 2011]. We explored both approaches, but found neither led to unbiased or even nearly unbiased inference (data not shown). For Zollner and Pritchard [2007], this failure is presumably down to the discrepancy between correcting for a *P* value that exceeds some threshold and selecting the SNP with the minimum *P* value. In the case of Sun et al. [2011], it is possible that single loci do not contain sufficient information for a bootstrap based correction; the corrected estimates tended to be biased in the opposite direction, suggesting the method was over‐correcting.

Our proposed solution is to use proportional testing and either avoid variable selection altogether by using the PCs that capture the majority of genetic variation in the region, or integrate over the variable selection using BMA. Either method maintains type 1 error, and the BMA approach appears more powerful than the PC approach, although both have reduced power compared to the hypothetical scenario of being able to test the causal variants themselves. Recall from equation (4) that the ppp depends on both the posterior probabilities of individual SNP models and the degree of departure from the null under each model, $P^{*}\left(m\right)$. If the posterior probability was spread broadly over the model space and $P^{*}\left(m\right)$ varied considerably across models, the distribution of ppp would be far from uniform, with too few observations in the tails. The fact that we observe only mildly conservative type 1 errors, even in the weakest associations considered here (case control sample sizes of 2,000, and relative risks of 1.1), probably reflects our requirement in simulating our datasets that some nominal level of association must be observed for at least one SNP in a region. This means that, for our simulated data, the posterior probabilities tend to be mainly focused on a small subset of models, and these models tend to contain sets of SNPs related by LD so that $P^{*}\;\left(m\right)$ does not vary substantially across the set of models on which most of the posterior probability is concentrated. Whether caution is needed in applying this approach in smaller datasets depends somewhat on a researcher's view. Certainly, it is unlikely that the ppp distribution would be uniform if small samples with weak associations were used, and therefore power to detect departure from colocalisation would be reduced. However, the alternative to colocalisation being tested is not just that of no colocalisation, but implicitly that of distinct causal variants in two traits. If there is weak or only limited evidence for association with either trait, then it does seem appropriate that one cannot reject a null of colocalising causal variants in favour of an alternative distinct causal variants. On the other hand, if evidence for association has been independently established, a researcher may be sure that there are causal variants for each trait although there is only weak evidence in the currently available datasets, in which case they may wish to explore calibrating the ppp distribution, for example by means of simulation [Hjort et al., 2006], so that its null distribution is uniform between [0, 1].

Regions are typically defined after GWAS studies according to genetic distance in order to describe the physical region within which a causal variant tagged by the association is expected to lie. T1DBase uses a definition of 0.1cM surrounding any single SNP with $P<5\times 10^{-8}$ and we used a 0.1cM window around the most associated SNP in our AITD analysis. If regions were defined more broadly, one might expect the BMA method to be relatively unaffected, as SNPs beyond the boundary of the current regions would not show much evidence for association. On the other hand, we expect the power of the PC approach would decrease, as the number of components, and therefore the degrees of freedom of the test, would increase without any compensatory increase in information.

We have proposed alternative approaches to colocalisation testing, but how should a researcher choose that approach and the parameters specific to that approach? Our view is that we would use PCs as a first pass if many loci are under consideration, to prioritise regions for more detailed analysis. The optimal number of components is unknown, but a number that captures something in the region of 70–90% of genetic variation seems acceptable. However, if evidence for association is strong in both datasets, BMA is a more powerful approach and there seems little reason beyond computational expense to prefer averaging over the universe of two SNP models to that of three SNP models. However, in large regions, a relatively modest number of SNPs can cause the number of three SNP models to be infeasible. For example, 100 SNPs can generate 4,950 two SNP models but 161,700 three SNP models. We would not suggest exploring four SNP models because that seems likely to spread the posterior probabilities very thinly, and we haven't explored combining two and three SNP models because it is unclear what prior weight should be given to a two SNP compared to a three SNP model.

As an example application of our proposed approaches, we analysed 13 loci associated with the autoimmune thyroid diseases GD and HT. Reassuringly, inference was broadly similar regardless of method, and we and showed that in the seven regions where a locus has been associated with the two diseases, the data are generally consistent with common causal variants exerting an equal effect on each disease. In regions previously significantly associated with only GD, posterior predictive *P* values are unlikely to detect any departure from colocalisation for reasons described above, but here the Bayesian approach does allow us to use make some inference using the posterior distribution of η. Given the relatively smaller number of HT cases (462) compared to GD (2,285), it might be expected that many of the loci only associated with GD have failed to be reach significance for HT due to lack of power. Estimates of power to detect association with HT under the assumption of equal effects in GD and HT are broadly similar across the seven regions associated with both diseases and the six regions associated with GD only (Supplementary Table S1), but these are likely to be over optimistic due to the expected bias in the GD effect size estimates. However, for five of these six regions, the evidence suggests that failure to detect association with HT is more likely to be due to a discrepancy in effect size, with the effect considerably larger in, and possibly specific to GD, compared to HT, rather than a lack of power. However, while the data suggest that *LPP* may have a stronger effect on GD compared to HT, and that *CCR6* may be an undetected HT locus, the small sample size for HT prevents us from drawing this conclusion with any confidence.

There is a pressing need for more widespread use of formal colocalisation testing. Researchers are turning to eQTL data to interpret GWAS results by simply considering whether an eQTL SNP is associated with any disease [Nicolae et al., 2010], visually [Trynka et al., 2011], by conditional testing [Nica et al., 2010], by naive application of a proportional colocalisation test [Plagnol et al., 2009; Wallace et al., 2012] or by attempting to integrate disease and gene expression association signals in networks [Hao et al., 2012]. Where colocalisation tests have been naively applied, we expect the null hypothesis of colocalisation has been rejected too readily, although this will affect loci with small and moderate effects to a greater degree than those with large effects. Thus, for our earlier analysis of colocalisation between T1D and monocyte gene expression signals [Wallace et al., 2012], the list of loci compatible with colocalisation are likely correct, but some loci were probably erroneously rejected, and re‐analysis of these data will be required.

In the case of network analysis, results can be difficult to reconcile with simple representations of the data. For example, integration of lung expression and asthma genetic association data led to the identification of *GSDMA* as the most likely causal gene for asthma in the 17q21 region [Hao et al., 2012], despite a graphical representation of the data showing that the SNPs most strongly associated with *GSDMA* expression were relatively weakly associated with asthma, and, *vice versa*, that the SNPs most strongly associated with asthma showed relatively weaker levels of association with *GSDMA* expression compared to the strongest signals. The asthma association on the 17q21 region was one of the first cases of explicitly using expression data to interpret disease association, with the association with asthma initially attributed to *ORMDL3* based on expression data from EBV transformed cell lines [Moffatt et al., 2007] and subsequently to *GSDMB* from a reanalysis of the same data [Moffatt et al., 2010]. Candidate gene hypotheses have been constructed for all three genes. The lung expression data have greater potential for revealing the underlying gene, but, to hold confidence in results of analyses, particularly when the results contrast with simple visual inspection of the data, requires careful examination of the properties of the statistical method used.

Given the tissue‐specific nature of many eQTLs identified to date [Dimas et al., 2009; Fairfax et al., 2012], there is a need for more large, publicly available eQTL datasets in a variety of disease relevant tissues and purified cell subsets to support the interpretation of existing GWAS data. Although expression data is typically shared after the publication of an eQTL study, we note that the genetic data must also be made available to allow full integration of eQTL and disease signals at shared loci. The increasing abundance of substantial GWAS datasets and the increasing availability of large eQTL datasets [Fairfax et al., 2012; Fu et al., 2012; Hao et al., 2012], together with our reassurance that it is possible to conduct these tests whilst maintaining type 1 error rates and the availability of software in our R package will facilitate more widespread formal colocalisation testing. Integration of genetic association data has the potential to refine understanding of underlying genetic mechanisms and aid in the design of follow‐up studies already underway.

## Conflict of Interest

The author has no conflict of interest to declare.

## Supplementary Material

Disclaimer: Supplementary materials have been peer‐reviewed but not copyedited.

Supporting Information
