# Supplementary material for: Statistical Testing of Shared Genetic Control for Potentially Related Traits
Source: Genet Epidemiol. 2013 Nov 5;37(8):802–13. doi: 10.1002/gepi.21765 (PMC4158901; doi:10.1002/gepi.21765)
Supplement: Supplementary file 1 — Supporting Information [file gepi-37-802-s6.zip › genepi-sup.pdf]

# Unbiased statistical testing of shared genetic control for potentially related traits: Supplementary Information

Chris Wallace

## Simulation

Once a "causal variant" SNP, $S$, was selected, control haplotypes were sampled randomly and case haplotypes sampled conditional on the allele carried at $S$. For a disease model with relative risk $r$, and given the minor (risk) allele at $S$ has frequency $\pi_0$, in controls, the frequency in cases is

$$\pi_1 = \frac{r\pi_0}{1 - \pi_0 + r\pi_0}.$$

Therefore when sampling case haplotypes, we over-sample haplotypes carrying the risk allele and under sample those carrying the protective allele by using sampling probabilities proportional to

$$P_S = \begin{cases} \frac{\pi_1}{\pi_0} & \text{haplotype carries risk allele} \\ \frac{1-\pi_1}{1-\pi_0} & \text{haplotype carries protective allele allele.} \end{cases}$$

For eQTL data, we simulated a response variable, $Y$ as a mixture of Gaussians

$$Y = \sqrt{0.7}Z + \sqrt{0.3}X$$

where $Z$ was sampled from a standard Gaussian and $X$ is the count of the minor allele at the causal SNP. Thus, $X$ would explain 30% of the variance of $Y$, or 30% of the simulated eQTL, independent of minor allele frequency.

## The effect size at a selected SNP

To calculate the bias in figure 2, we compared the estimated effect size at the sampled SNP to the true effect *at that SNP*, ie not at the causal SNP. If the causal SNP is $S$ and the selected SNP is $T$, then the underlying relative risk at $T$ is simplest to calculate in a haploid system, which is equivalent to assuming Hardy Weinberg equilibrium. Given

$$\rho' = \rho(S,T)\sqrt{\pi_S \pi_T (1-\pi_S)(1-\pi_T)}$$

where $\rho(S,T)$ is the correlation between $S$ and $T$, then the expected proportion of cases in the population conditional on the allele carried at SNP $T$ is

$$\begin{cases} D_1 = \dfrac{r(\pi_S \pi_T + \rho') + ((1-\pi_S)\pi_T - \rho')}{\pi_T} & T \text{ is risk allele} \\ D_0 = \dfrac{r(\pi_S(1-\pi_T) - \rho') + ((1-\pi_S)(1-\pi_T) + \rho')}{1-\pi_T} & T \text{ is protective allele} \end{cases}$$

and the relative risk is $\dfrac{D_1}{D_0}$. For a rare disease such as T1D, relative risks and odds ratios are approximately equal.

## Implementation of Bayesian Model Averaging

Bayesian model averaging requires evaluating all possible multiple SNP models in each trait, and conducting colocalisation testing for each model. We began by defining the posterior probability of model $j$ for both traits as,

$$\pi_j = \frac{\pi_j^1 \pi_j^2}{\sum_k \pi_k^1 \pi_k^2}$$

where $\pi_j^i$ is the posterior probability of model $j$ for trait $i$ and a model $j$ indicates which SNPs are included in the model. Even when the number of SNPs to be tested is fixed at two, the number of possible models is $\frac{p!}{2!(p-2)!}$. Whilst testing all models is feasible if computationally expensive for analysis of real data, it is impractical for simulations. To reduce the computation burden, we first evaluated all $p$ single SNP models and indentified the set of SNPs with very low posterior probability ($\pi_j < 0.01$). We then excluded any two SNP model containing *only* SNPs from this set. If $p_0 < p$ such SNPs were identified, this reduced the number of models to test to $\frac{p!}{2!(p-2)!} - \frac{p_0!}{2!(p-2)!}$.

For the purposes of simulation, we used the profile likelihood approach to generate a $\chi^2_1$ distributed test statistic and averaged the resulting $p$ values, $P_j$, over the model space to calculate an overall posterior predictive $p$ value, $\sum_j P_j \pi_j$. For the application to AITD, we integrated the $p$ value associated with the $\chi^2_2$ distributed test statistic calculated assuming $\eta$ was known over both the posterior distribution of $\eta$ given each model, and the posterior of the model space. The latter is formally correct, but comptationally too expensive for simulation, and the profile likelihood $p$ value and the posterior predictive $p$ value have been shown to be very similar for large samples.

| Region | SNP | MAF | GD | | HT | | Power, $\alpha =$ | |
|---|---|---|---|---|---|---|---|---|
| | | | OR | p value | OR | p value | 0.05 | $10^{-6}$ |
| *Associated with GD and HT in published study* | | | | | | | | |
| 1p13.2/*PTPN22* | rs2476601 G>A | 0.096 | 1.55 | $4.03 \times 10^{-16}$ | 2.02 | $3.74 \times 10^{-15}$ | 0.99 | 0.37 |
| 2q33.2/*CTLA4*/*ICOS* | rs11571297 G>A | 0.493 | 0.72 | $2.81 \times 10^{-23}$ | 0.82 | $3.21 \times 10^{-3}$ | 0.99 | 0.490 |
| 2p25.1/*TRIB2* | rs1534422 A>G | 0.455 | 1.16 | $4.69 \times 10^{-6}$ | 1.24 | $1.64 \times 10^{-3}$ | 0.61 | 0.004 |
| 3q27.3/3q28/*LPP* | rs13093110 C>T | 0.452 | 1.18 | $8.17 \times 10^{-7}$ | 1.20 | $7.09 \times 10^{-3}$ | 0.70 | 0.008 |
| 6q15/*BACH2* | rs72928038 G>A | 0.177 | 1.21 | $3.63 \times 10^{-6}$ | 1.30 | $1.36 \times 10^{-3}$ | 0.64 | 0.005 |
| 10p15.1/*IL2RA* | rs706779 A>G | 0.467 | 0.85 | $2.27 \times 10^{-6}$ | 0.84 | 0.0125 | 0.674 | 0.007 |
| 11q21/– | rs4409785 T>C | 0.173 | 1.21 | $5.37 \times 10^{-6}$ | 1.34 | $3.54 \times 10^{-4}$ | 0.63 | 0.004 |
| *Associated with GD only in published study* | | | | | | | | |
| 1p36.32/*TNFRSF1* | rs2843403 C>T | 0.362 | 0.84 | $7.94 \times 10^{-7}$ | 0.97 | 0.696 | 0.69 | 0.007 |
| 1q23.1/*FCRL3* | rs7522061 T>C | 0.480 | 1.16 | $1.08 \times 10^{-5}$ | 1.03 | 0.634 | 0.60 | 0.004 |
| 6q27/*CCR6* | imm_6_167338101 A>C | 0.408 | 0.84 | $3.30 \times 10^{-7}$ | 0.88 | 0.056 | 0.71 | 0.009 |
| 12q12/*PRICKLE1* | rs4768412 C>T | 0.363 | 1.19 | $3.30 \times 10^{-7}$ | 1.00 | 0.949 | 0.73 | 0.010 |
| 14q31.1/*TSHR* | rs2300519 T>A | 0.380 | 1.54 | $1.34 \times 10^{-38}$ | 0.93 | 0.295 | 1 | 0.95 |
| 16p11.2/*ITGAM* | rs57348955 G>A | 0.396 | 0.83 | $3.76 \times 10^{-8}$ | 0.91 | 0.188 | 0.76 | 0.013 |

Supplementary Table 1: **Power to detect association with Hashimoto's Thyroiditis to confirmed loci for Graves' Disease.** Region denotes chromosomal region and most likely candidate gene(s) where available [9]. GD=Graves' Disease; HT=Hashimoto's Thyroiditis; MAF=minor allele frequency in controls.

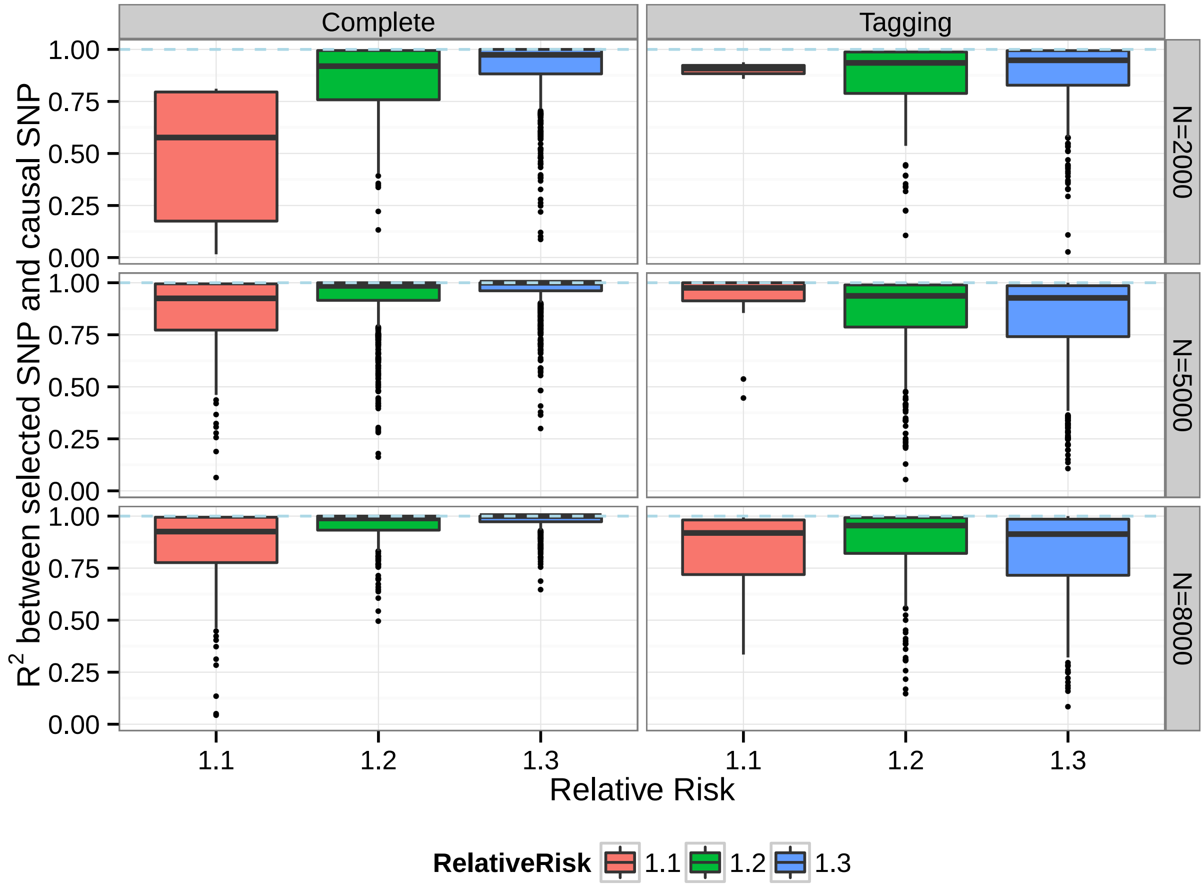


Supplementary Figure 1: **The most associated SNP in a region is not necessarily the causal SNP.** Boxplots show the distribution of $r^2$ between the SNP with the smallest $p$ value (conditional on $p < 1 \times 10^{-8}$) and the causal SNP from simulated data, either under tagging or complete genotype coverage. Increasing the effect size increases the range of tagging SNPs detectable, and hence can have the apparently counter-intuitive result of decreasing the correlation between selected and causal SNPs. However, if complete genotype coverage is available, the LD between selected and causal SNPs tends to increase with effect size or sample size.

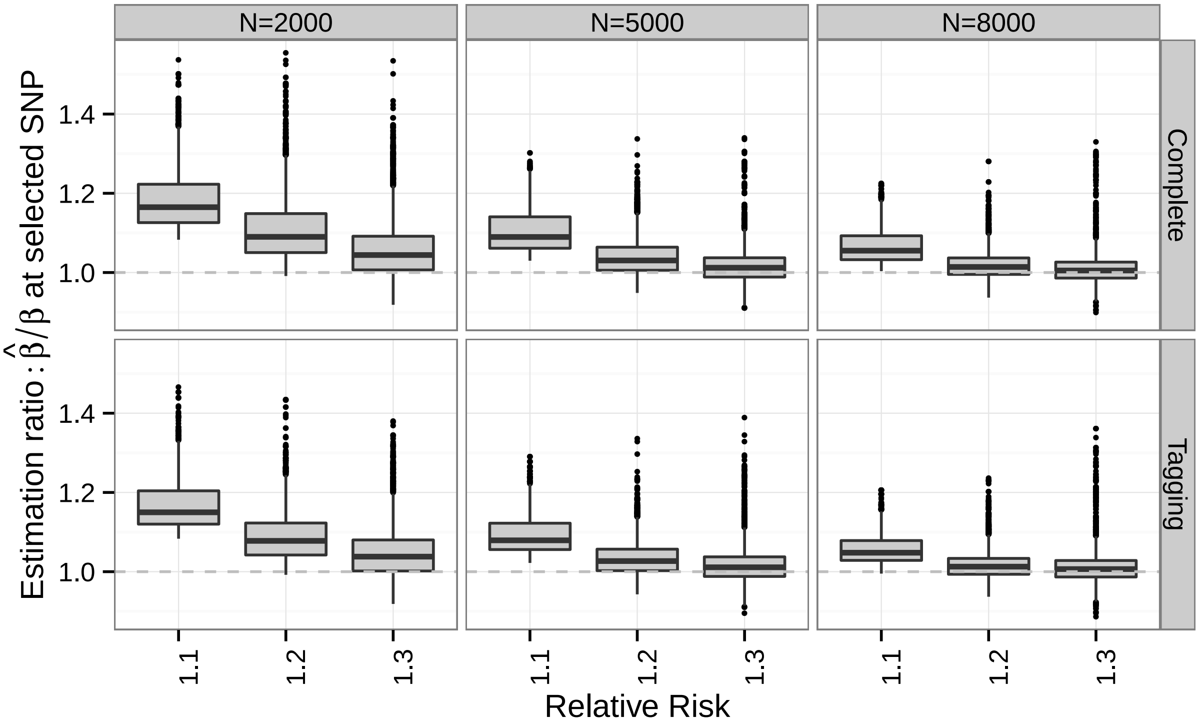


Supplementary Figure 2: Effect sizes at selected SNPs tend to be overestimated. Boxplots show the distribution of the ratio of the estimated effect size ($\hat{\beta}$) to the true effect size ($\beta$) at the most associated SNP in a region. Simulations were conducted for samples of N cases and N controls, with a relative risk at a randomly selected “causal SNP” of 1.1, 1.2 or 1.3, under either a complete genotyping scenario (all SNPs in 1000 Genomes, top row) or the subset of SNPs appearing on the Illumina Human Omni Express chip chip (“Tagging”, bottom row). Estimated effects are more likely to be biased for smaller effect sizes and sample sizes.

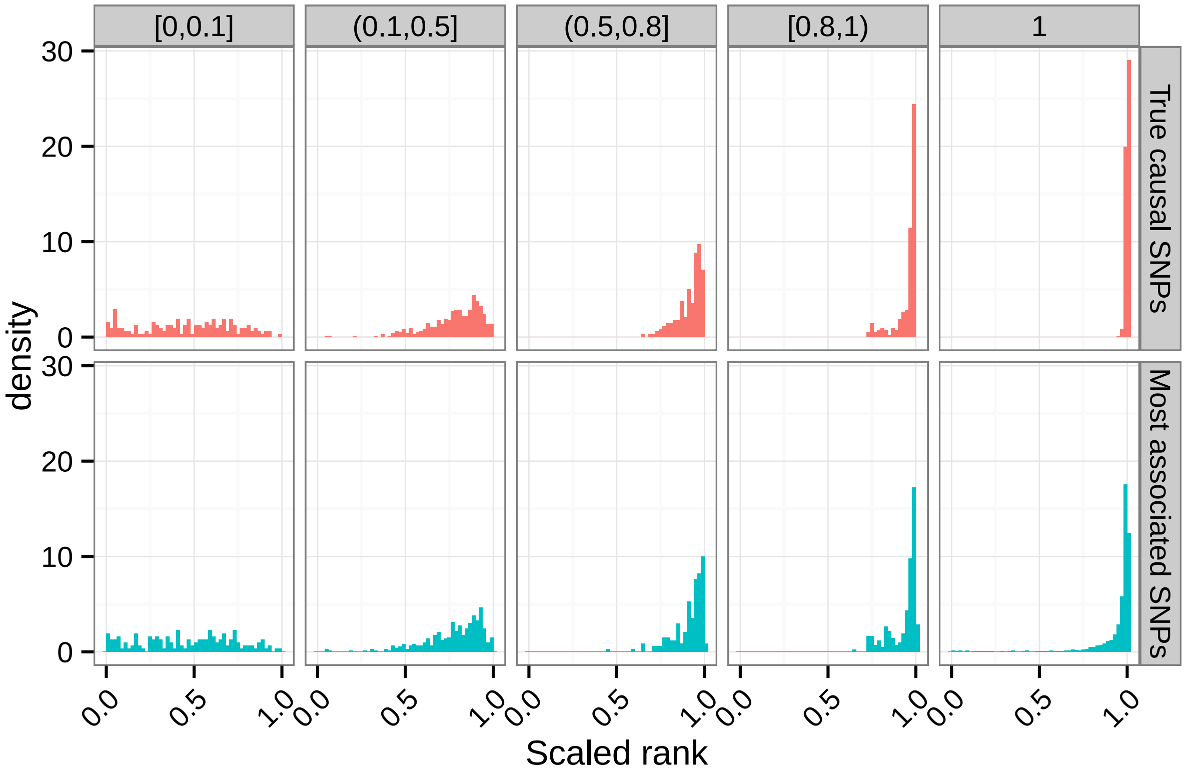


Supplementary Figure 3: **Distribution of Nica *et. al*'s rank statistic.** The statistic is evenly distributed within [0,1] when the LD between the causal variants is negligable, but is increasingly biased towards 1 as the LD increases. Columns are divided by the $r^2$ between distinct causal variants, with $r^2 = 1$ indicating a shared causal variant. The top row shows the optimal result that could be obtained if conditioning on the true causal variant were possible, the bottom row shows the effect of conditioning on the most associated SNP is to reduce the degree of skew. Results are shown for a complete genotyping scenario, with a sample size of 2000 and a relative risk of 1.3. Similar effects are seen under tagging or complete genotyping approaches, but the skew towards 1 occurs more rapidly with larger samples and effect sizes.

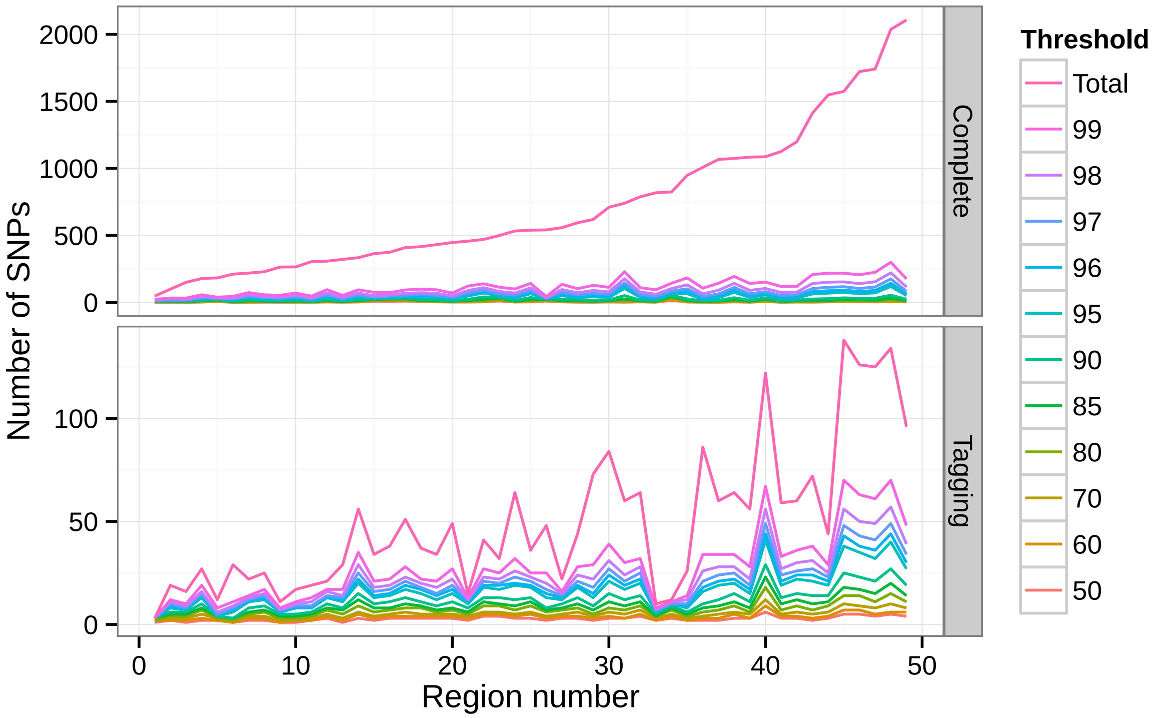


Supplementary Figure 4: **The number of principal components required to capture a predefined proportion of the variance.** The 49 regions used for simulation are displayed, unlabelled and ordered by the total number of SNPs. The majority of variation can be captured by a relatively modest number of components even for regions containing large numbers of SNPs. Threshold specifies the minimum proportion of variance captured, or “Total” for the total number of SNPs in a region.

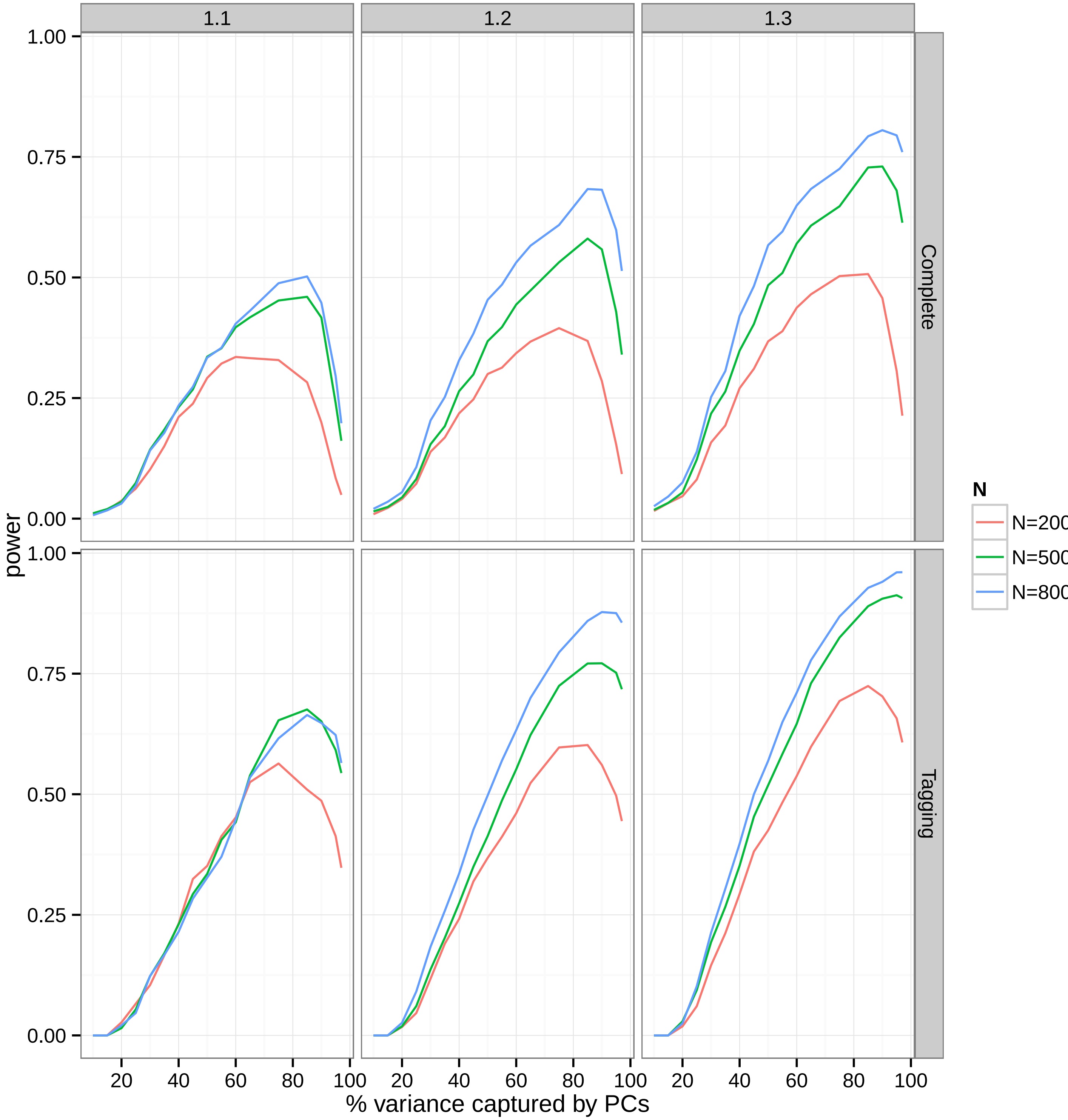


Supplementary Figure 5: **Power using colocalisation testing of principal components according to the proportion of genotype variance captured.** Power is shown for all simulated datasets where the $r^2$ between the causal SNPs was less than 0.5.

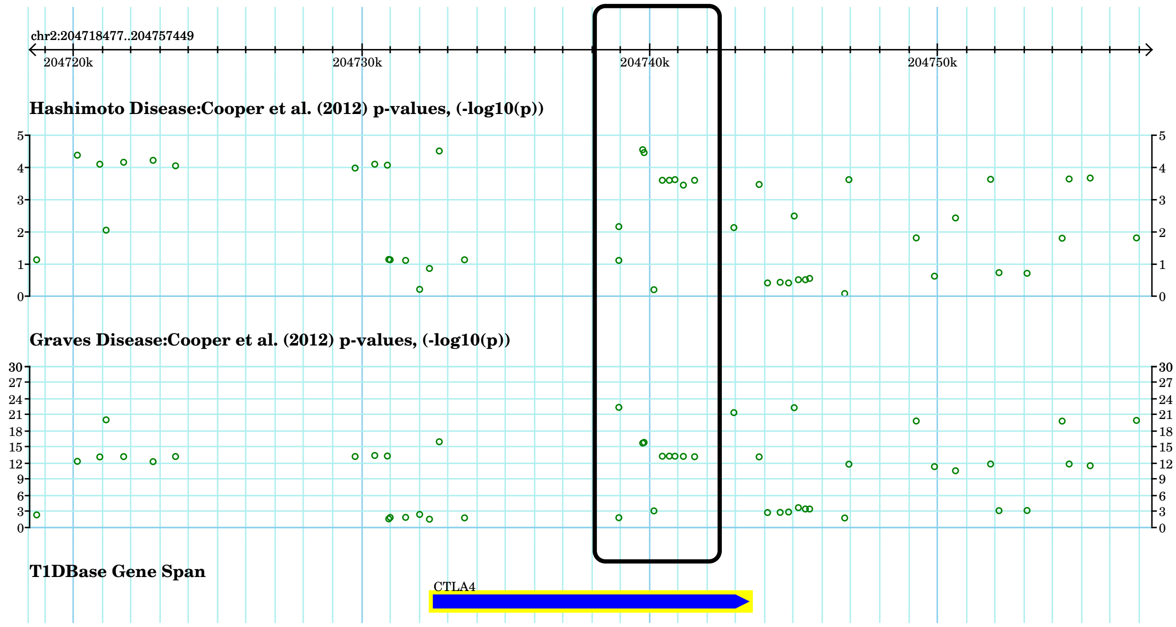


Supplementary Figure 6: Single SNP p values for GD and HT in the *CTLA4/ICOS* region on 2q33.2. A red box highlights the region of most obvious difference.
